# Modified de Gramont with oxaliplatin in the first-line treatment of advanced colorectal cancer

**DOI:** 10.1038/sj.bjc.6601237

**Published:** 2003-09-30

**Authors:** M S Braun, F Adab, C Bradley, K McAdam, G Thomas, N J Wadd, D Rea, R Philips, C Twelves, J Bozzino, C MacMillan, M P Saunders, R Counsell, H Anderson, A McDonald, J Stewart, A Robinson, S Davies, F J Richards, M T Seymour

**Affiliations:** 1 Cancer Research UK Centre in Leeds, Cookridge Hospital, Leeds LS16 6QB, UK; 2 Staffordshire Oncology Centre, Royal Infirmary, Princes Road, Hartshill, Stoke on Trent ST4 7LN, UK; 3 Department of Oncology, Bradford Royal Infirmary, Duckworth Lane, Bradford BD9 6RJ, UK; 4 Department of Clinical Oncology, Peterborough District Hospital, Thorpe Road, Peterborough, PE3 6DA, UK; 5 Department of Oncology, Derbyshire Royal Infirmary, London Road, Derby DE1 2QY, UK; 6 Department of Oncology, South Cleveland Hospital, Marton Road, Middlesbrough TS4 3BW, UK; 7 CRC Institute for Cancer Studies, Vincent Drive, Edgbaston, Birmingham B15 2TT, UK; 8 Department of Oncology, Charing Cross Hospital, Fulham Palace Road, London W6 8RF, UK; 9 Beatson Oncology Centre, Western Infirmary, Glasgow G11 6NT, UK; 10 Northern Centre for Cancer Treatment, Newcastle General Hospital, Westgate Road, Newcastle upon Tyne NE4 6BE, UK; 11 Northamptonshire Centre for Oncology, Northampton General Hospital, Biling Road, Northampton NN1 5BD, UK; 12 Christie Hospital, Wilmslow Road, Withington, Manchester M20 4BX, UK; 13 Gloucestershire Oncology Centre, Cheltenham Royal Infirmary, Sandford Road, Cheltenham GL53 7AN, UK; 14 Department of Oncology, Southend Hospital, Westcliffe-on-Sea, Essex SS0 0RY, UK

**Keywords:** fluorouracil, oxaliplatin, colorectal carcinoma

## Abstract

We previously reported high activity for oxaliplatin and a modified de Gramont regimen (OxMdG) in a single centre study of patients with metastatic colorectal cancer. We now report results with a further 56 patients treated at 14 centres. Low rates of grade 3 and 4 toxicity were seen, with no toxic deaths. Objective response rates were CR/PR=53%; NC=34.7%; PD=12.2%. Median time to progression was 8.3 months and overall survival was 14.5 months. This regimen is more convenient than those based around the conventional de Gramont regimen but is highly active and well tolerated; it forms part of a current UK MRC phase 3 trial.

The fortnightly infusional LV5FU2 or ‘de Gramont’ regimen of fluorouracil (FU) and leucovorin (LV) is less toxic than bolus FU/LV, but gives a higher response rate and longer progression-free survival (de Gramont *et al*, 1997). It has therefore been used as the partner for other drugs in combination regimens (de Gramont *et al*, 2000; Douillard *et al*, 2000). ‘FOLFOX4’–the combination of LV5FU2 with oxaliplatin 85 mg m^−2^–was recently compared to the US standard bolus irinotecan, FU and folinic acid regimen (IFL) in NCCTG trial N9741, and showed improved survival and toxicity (Goldberg *et al*, 2002). It is therefore now accepted by the FDA as a standard comparator regimen for licensing trials in advanced colorectal cancer.

Regimens based on LV5FU2 are, however, time consuming, cumbersome and expensive (Ross *et al*, 1998). Their benefits are, therefore, partly offset by the demands that they place on patients and the health-care system. Two strategies are being investigated to overcome these problems (1) to replace the complex 2-day sequence of FU and LV infusions and injections with a simplified infusion administered at home using a portable pump; or (2) to replace LV5FU2 altogether, using the oral prodrug, capecitabine.

We recently published pilot data on an ‘Oxaliplatin + Modified de Gramont’ regimen (OxMdG). In our single centre study, this new regimen demonstrated exceptional activity as first-line treatment, and was also well tolerated and convenient (Cheeseman *et al*, 2002). We now present data from a prospective multicentre phase 2 study of a further 55 patients receiving the same OxMdG schedule as first-line treatment for advanced colorectal cancer.

## PATIENTS AND METHODS

Following Multicentre Research Ethics Committee approval, we recruited 56 patients at 14 institutions between January 2000 and July 2001. All patients had inoperable metastatic colorectal cancer and had not received prior chemotherapy for metastatic disease. Other eligibility criteria were: WHO performance status 0–2; bilirubin <50 *μ*moll^−1^l; ALP and transaminases <3 × upper limit of normal; WBC>3 × 10^9^ l^−1^; neutrophils >1.5 × 10^9^ l^−1^; platelets>100 × 10^9^ l^−1^; GFR (Cockcoft estimate or EDTA clearance) >60 ml min^−1^. Contraception was required for women of child-bearing potential. Written informed consent was obtained from all patients prior to study entry.

### Treatment

Semipermanent venous access was established with a single-lumen Hickman line, Portacath, or PICC line according to local practice. Low-dose anticoagulation with warfarin (10 mg on the day of insertion and 1 mg daily thereafter) was recommended (Bern *et al*, 1990). The OxMdG regimen was given every 14 days as follows.

Intravenous bolus dexamethasone 8 mg and granisetron 1 mg were given prior to chemotherapy. Then, oxaliplatin 85 mg m^−2^ was given concurrently with *l-*leucovorin 175 mg (flat-rate), via a *Y*-connector, as a 2 h i.v. infusion. Each drug was diluted in 250 ml 5% dextrose, to avoid mixing oxaliplatin with saline. Next, FU was given at 400 mg m^−2^ i.v. bolus over 5 min, followed by 2400 mg m^−2^ as a 46 h FU infusion using a disposable elastometric pump (Baxter LV5®) or equivalent device. Oral dexamethasone 4 mg t.d.s. was given on day 2, b.d. on day 3 and o.d. on day 4.

Treatment was initiated in the chemotherapy day-unit and continued at home. After the infusion the line was flushed by the patient's community nurse; Hickman and PICC lines were also flushed weekly between treatments. Where necessary, prior to establishing central venous access, the 46 h FU infusion was given in 2000 ml saline via a peripheral cannula, as an inpatient.

### Evaluation of response and duration of treatment

Blood tests and clinical evaluation were performed each fortnight, prior to treatment. Toxicity was evaluated using NCI CTC criteria (version 2). In particular, patients were monitored for peripheral sensory symptoms. If patients developed paraesthesia which persisted for 14 days after oxaliplatin administration, became painful, or caused functional impairment, oxaliplatin was omitted from the schedule but the other drugs were continued. Other dose adjustments followed standard guidelines: doses of both cytotoxic drugs were reduced by 20% after grade 3–4 toxicity, or after 2 delays for grade 2 toxicity.

Tumour marker assays were repeated every 4 weeks and CT scans (or other relevant imaging) every 12 weeks. The response to chemotherapy was assessed by RECIST criteria (Therasse *et al*, 2000), except that confirmatory scans 4 weeks after a response were not required.

The plan was to deliver six treatment cycles at 2-week intervals followed by response evaluation, then a further six cycles for patients with stable or responding disease. Treatment beyond 12 cycles could be offered the clinician's discretion, but this was not normal practice in most participating institutions.

## RESULTS

### Treatment delivery and toxicity

In all, 56 patients were registered, but one did not receive chemotherapy because of clinical deterioration soon after registration. Baseline characteristics of the 55 patients treated are summarised in Table 1

**Table 1:** Patient characteristics

Number	55
Sex (m : f)	38 : 17
Age: median (range) (years)	59 (23–79)
*Performance status*	
0	24 (43.6%)
1	28 (50.9%)
2	3 (5.4%)
*Primary site*	
Colon	28^a^
Rectum	27
*Previous treatment*	
Adjuvant 5FU/FA	14
Pre- or post-op radiotherapy	8
Marimastat	1

a Including one appendiceal carcinoma.

; all but three had measurable disease by RECIST criteria.

A total of 479 cycles of OxMdG were delivered (median 10 cycles per patient). In all, 62 (13%) of these cycles were delayed for toxicity, most commonly haematological: 36 (8%) for neutropenia and 21 (4%) for thrombocytopenia. Only three cycles (<1%) were delayed for diarrhoea, three cycles for venous line infection and one because of neutropenic sepsis. In one patient the cycle was extended to three-weekly (out-with protocol) because of persistent low blood counts.

Five patients (9%) required omission of oxaliplatin from the schedule for sensory symptoms occurring at cycles 7–11, and one patient (2%) because of an allergic reaction at cycle 9. In all, 25 (45%) patients underwent dose reduction at some point during their treatment, most commonly because of grade 3 neutropenia or thrombocytopenia.

Toxicity per cycle (NCI CTC, version 2) occurring during the first six cycles is shown in Table 2

**Table 2:** Toxicity per cycle of OxMdG, cycles 1–6

	**All grades (%)**	**Grade 3–4 (%)**
Lethargy	95 (31)	9 (3)
Nausea	45 (14)	3 (1)
Vomiting	30 (10)	4 (1)
Diarrhoea	86 (28)	7 (2)
Mucositis	52 (17)	1 (<1)
Skin (HFS)	43 (14)	0
Neuropathy	167 (55)	0
Neutropenia	36 (12)	9 (3)
Thrombocytopenia	64 (21)	0

*N*=304 cycles.

. The maximum haematological toxicity experienced at any point in treatment was grade 3 or 4 in 20% patients and grade 2 or less in the remaining 80% patients. No grade 4 non-haematological toxicity was seen. The maximum non-haematological toxicity experienced was grade 3 in 16 patients (29%), although in 6 cases this was lethargy, scoring of which is subjective. Five (9%) patients experienced grade 3 diarrhoea, three (5%) had grade 3 nausea or vomiting and one (2%) had grade 3 mucositis. Overall, 23 patients (42%) experienced a grade 3–4 haematological or non-haematological event at some point in treatment.

Grade 1 or transient grade 2 neuropathy was common, occurring in 55% of patients during the first six cycles, but at some point in treatment in 78% of patients.

### Antitumour activity

In all, 52 patients had measurable disease at the outset. Three were withdrawn from the trial: one declined further treatment after cycle 2 following an episode of *Campylobacter* sp. diarrhoea, one was withdrawn after cycle 2 because of a pelvic abscess requiring surgery; the third after cycle 4 because of an ischaemic leg requiring embolectomy, which was complicated by a postoperative myocardial infarct. These three patients are not included in the response rate analysis, but are included in the intent-to-treat survival analysis.

Of the remaining 49 patients, four had a radiological complete response (8.1%) and 22 (44.9%) a partial response, giving an overall response rate of 53%. A further 17 (34.7%) had stable disease for at least 12 weeks, so a total of 87% of patients had some evidence of anticancer activity. Only six (12.2%) patients had disease progression as their only response.

Apart from the planned end of treatment, the reason for stopping treatment was given as progressive disease in 13 patients and toxicity in six (usually grade 3 lethargy).

### Survival

**Figure 1:**
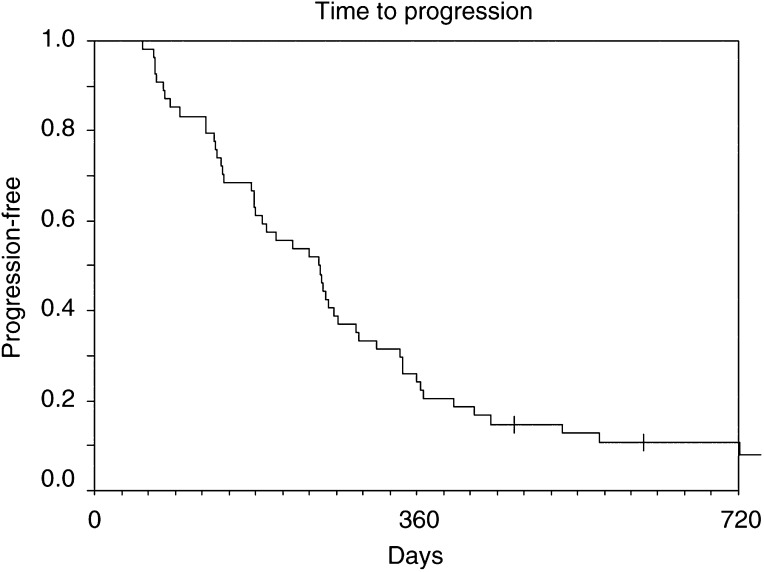
Time to progression, all patients.

Time to progression (TTP) and overall survival (OS) were calculated for all 55 patients (Figures 1

and 2

**Figure 2:**
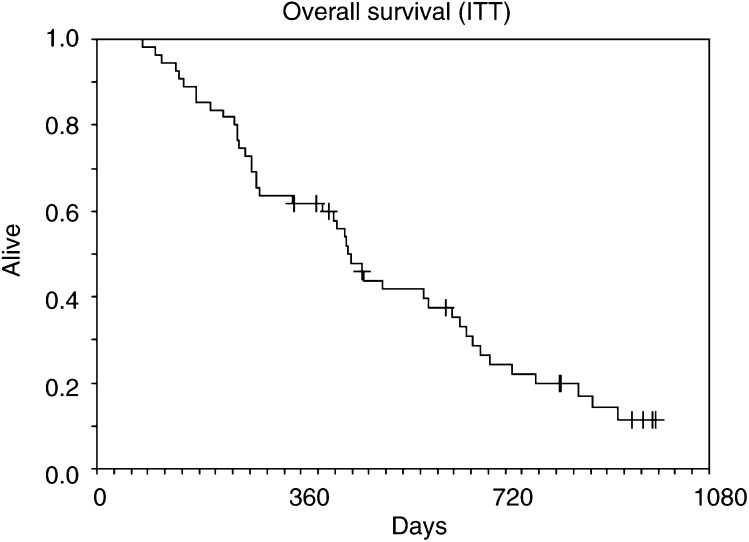
Overall survival, all patients.

). Median TTP was 8.3 months (range 1.8–29.8 months) and median OS was 14.5 months (range 2.6–31.1 months). In all, 62% of patients were alive 1 year after registration.

In total, 25 (45%) patients received further anticancer treatment after completion of the trial protocol. Four underwent resection of metastases: two hemihepatectomies, one resection of abdominal wall disease and one resection of bilateral Krukenberg tumours. One of the patients undergoing liver surgery was found to have had a pathological complete response. In all, 10 (18%) patients received radiotherapy, and 22 (40%) received second-line chemotherapy, usually including mitomycin or irinotecan.

At 18 months after study closure, and with median 35 months potential follow-up, 11 (20%) patients remain alive. In 43 patients the cause of death was progressive disease; the remaining patient had a pulmonary embolism within 30 days of the final cycle of treatment.

## DISCUSSION

We recently reported a single-institution phase II trial of OxMdG in 62 patients with advanced colorectal cancer. That trial included 24 assessable patients who received the regimen as first-line therapy for metastatic colorectal cancer, whose case-notes and scans were reviewed by an independent external radiologist and oncologist; 72% had RECIST complete or partial responses, confirmed by a second scan; a further 8% had partial responses that were not confirmed by a second scan (Cheeseman *et al*, 2002). That high response rate (72% confirmed; 80% confirmed+unconfirmed) was markedly higher than, although statistically consistent with, other first-line trials of FU/FA with oxaliplatin (De Gramont *et al*, 2000;Tournigand *et al*, 2001; Goldberg *et al*, 2003). Nevertheless, this high response rate was achieved in a single centre trial; also, we had some concerns that, despite low rates of grade 3 and 4 toxicity, there were two (3.2%) treatment-related deaths.

The current trial was, therefore, initiated to see whether the high activity of the regimen could be reproduced in a multicentre setting, and to further investigate its safety prior to use in a phase III trial. We have confirmed that OxMdG is well-tolerated, with extremely low levels of grade 3 and 4 toxicity, and no deaths were attributable to chemotherapy. The one death not attributed to disease progression occurred after the discontinuation of treatment but within 30 days of the last cycle, and was due to a pulmonary embolism. Importantly, although the objective response rate of 53% is lower than in our previous study, this has been achieved in a larger group of patients as part of a multicentre study. Moreover, this is in line with other experiences as shown in Table 3

**Table 3:** Comparison of FOLFOX and OxMdG regimes

	**FOLFOX 4 (De Gramont *et al*, 2000)**	**FOLFOX 6 (Tournigand *et al*, 2001)**	**OxMdG (Cheeseman *et al*, 2002)**	**OxMdG (this report)**
Total FU dose/cycle (bolus+infusion)	800+1200	400+2400 (−3000)	400+2400	400+2400
Total oxaliplatin dose/cycle	85	100	85	85
Total colorectal patients	210	113	25	55
Assessable for response	207	109	24	52
Response rate CR+PR	50.7	56	72	53
Median FFS (months)	8.2	8.9	10.6	8.3
Median OS (months)	16.2	Not reported	16.7	14.5

which summarises the results of our previous study, those from FOLFOX4 (de Gramont *et al*, 2000) and FOLFOX6 (Tournigand *et al*, 2001).

These data suggest that OxMdG is a more convenient but equally efficacious and well tolerated alternative to FOLFOX4, although a direct randomised comparison of the two regimens has not been made. OxMdG is now being further evaluated in the ongoing MRC CR08 (FOCUS) trial, which compares its use as first-line therapy or reserved for second-line, and includes a comparison with an equivalent irinotecan-containing regimen.
